# Machine learning-based estimation of structure-specific load around the ankle and knee joint during running using IMU data

**DOI:** 10.3389/fbioe.2026.1710980

**Published:** 2026-02-11

**Authors:** Sieglinde Bogaert, Jesse Davis, Benedicte Vanwanseele

**Affiliations:** 1 Human Movements Biomechanics Research Group, Department of Movement Sciences, KU Leuven, Leuven, Belgium; 2 KU Leuven Institute of Sports Science (LISS), KU Leuven, Leuven, Belgium; 3 Department of Computer Science, Leuven.AI, KU Leuven, Leuven, Belgium

**Keywords:** IMU, joint contact force, machine learning, musculoskeletal load, running, structure-specific load, tendon force

## Abstract

Running imposes substantial repetitive loads on the musculoskeletal system, which can lead to running-related overuse injuries. Therefore, effective structure-specific load management is essential for both prevention and rehabilitation. However, the conventional method for estimating structure-specific load (SSL) is time intensive to execute, and the resulting data is computationally expensive to analyze. This study aims to estimate the SSL on the Achilles and the patellar tendons, and the knee and ankle joint during running from data collected by one or two inertial measurement units (IMUs). We proposed a long-short-term-memory-based model that is trained and evaluated on a dataset of 43 participants. The estimated SSL during the stance phase of a running step achieved ${\text{R}}^{2}$ scores of 0.93, 0.84, 0.89, and 0.86 compared to the ground truth values for the Achilles and patellar tendon force, and ankle and knee contact forces, respectively. Furthermore, the results indicate that the SSL can be estimated practically and reliably during running using only time series data from a single pelvis-mounted IMU. These findings highlight the potential of using machine learning models applied to IMU data for SSL monitoring in real-world running scenarios.

## 1 Introduction

Running is a widely practiced physical activity with numerous health benefits, including a reduced risk of premature mortality, prevention of chronic diseases, and improved mental health (Lee et al., 2017; Pedisic et al., 2020). However, running imposes substantial repetitive loads on the musculoskeletal system, causing microstructural damage. When the cumulative damage surpasses a critical threshold, running-related overuse injuries (RROIs) may occur (Edwards, 2018). Cumulative damage scales with the number of loading cycles and the magnitude of load raised to a structure-specific power (e.g., nine for the Achilles tendon) (Edwards, 2018), making RROI risk highly sensitive to increases in load magnitude. Running-related overuse injuries are common, primarily affecting the knee and lower leg (Kakouris et al., 2021), with typical conditions including Achilles tendinopathy, medial tibial stress syndrome, patellofemoral pain syndrome, iliotibial band syndrome, and plantar fasciitis (Kakouris et al., 2021). Because load drives RROI development, effective load management is key to both its prevention and rehabilitation (Edwards, 2018).

Effective load management depends on accurately assessing the load experienced by the musculoskeletal system during running. Traditional load monitoring approaches, which typically rely on training volume metrics like distance or duration, provide only rough load estimates and fail to account for inter-individual and session-to-session variability in structure-specific loading. In this study, the term structure-specific load (SSL) refers to the forces acting on specific musculoskeletal structures, namely the Achilles tendon, patellar tendon, knee joint, and ankle joint. The term SSL is anatomical in scope, indicating that each load estimate corresponds to a particular tendon or joint. Factors such as running speed, terrain, fatigue (Apte et al., 2021), and cadence affect SSL, underscoring the need for continuous SSL measurement to advance understanding of injury mechanisms and refine load management strategies.

Estimating SSL typically relies on laboratory-based motion capture systems, instrumented treadmills, and musculoskeletal modeling (Delp et al., 2007). Although these methods offer high-precision measurements, they are expensive, labor- and computationally-intensive, and laboratory-bound. This poses a barrier to the continuous assessment of SSL in natural running settings. Therefore, machine learning (ML) has emerged as a promising tool to estimate SSL in real-world settings (Liew et al., 2023; Burton et al., 2021; David et al., 2024; Rasmussen et al., 2023).

Machine learning increasingly enables estimating complex biomechanical signals from simpler inputs (Dorschky et al., 2023). Previous research has demonstrated that machine-learning models can estimate kinematics and ground reaction forces from inertial-measurement-unit data (Wouda et al., 2018; Dorschky et al., 2023). Moreover, prior studies have shown that machine learning can replace computationally expensive musculoskeletal modeling by accurately estimating SSL from kinematic data, ground reaction forces, electromyography, or kinetic data (Liew et al., 2023; Burton et al., 2021; Zhang J. et al., 2022). Despite this progress, these approaches still depend on laboratory-collected data, restricting their real-world applicability.

Wearable sensors, such as inertial measurement units (IMUs), offer a practical alternative to laboratory-based measurement equipment by enabling data collection in natural running settings. While machine learning approaches have shown considerable promise in estimating SSL during running from IMU data, current research has limitations. For example, two recent studies (David et al., 2024; Rasmussen et al., 2023) estimated SSL during running based on simulated IMU data and it is unclear how applicable such findings will be to real IMU data. One study trained an elastic net or XGBoost model using up to two simulated IMUs and achieved high accuracy for patellar tendon force (correlation of 0.96), but lower accuracy for Achilles tendon force (correlation of 0.72) (Rasmussen et al., 2023). A subsequent study increased the number of simulated IMUs to four, improving Achilles tendon force estimation ( ${\text{R}}^{2}$ of 0.93) and maintaining similar performance for patellar tendon force ( ${\text{R}}^{2}$ of 0.92). Additionally, knee and ankle contact forces were estimated with good accuracy (average ${\text{R}}^{2}$ of 0.90) using a Kohonen self-organizing map (David et al., 2024). Although these results are promising, they rely on simulated IMU data, which may not capture the variability, noise, and complexity of real-world signals. This gap limits the applicability of these findings to real-life running scenarios.

Zangene et al. (2022) demonstrated the feasibility of estimating SSL using physical IMUs. They trained a fully-connected neural network on data from three physical IMUs to estimate knee contact forces, achieving a correlation of 0.79. However, the study included only ten male participants and therefore it is unclear how well this will generalize to broader and more diverse populations. Furthermore, this study estimates SSL during gait. Indeed, the predominant focus of existing literature on estimating SSL is concerned with activities other than running (Giarmatzis et al., 2020; Zangene et al., 2022; Burton et al., 2021). Although these SSL estimations during non-running activities contribute meaningfully within their respective contexts, estimating SSL during running is most relevant for preventing RROI, which is the underlying motivation of this study.

While machine-learning-based SSL estimation from IMU data has been studied, the ability to estimate SSL at several RROI-prone lower-limb structures during running using physical IMUs, capturing both continuous stance-phase profiles and discrete SSL characteristics, has not yet been demonstrated. Therefore, this study estimates the Achilles and patellar tendon force, and the knee and ankle contact force using physical IMU data collected during running. We pursue three objectives. First, we investigate whether a machine learning model can estimate SSL during running. More specifically, we are interested in estimating SSL during the stance phase of a running step and the ability to estimate three SSL characteristics: peak, impulse, and average loading rate. Second, we assess how different types of features influence model performance. Third, we assess whether a single-IMU model can match the performance of a dual-IMU model, aiming to determine the most practical IMU setup for real-world use.

## 2 Methods

### 2.1 Participants

Forty-three volunteers, 36 males and 7 females, with varying sports engagement, participated in the study. Adults aged 18–60 years were eligible for participation. Individuals were excluded if they had a body mass index over 30, any current injury to the lower limbs that could hinder running, or neurological conditions affecting normal walking patterns. The social and societal ethics committee at the KU Leuven approved this study (G-2022-5367-R4), and all participants provided written consent.

### 2.2 Data collection

Each participant completed an 11-min warm-up on a treadmill, consisting of walking and running. Following this, the participants ran with their own running shoes (to reflect real-world conditions) at 2.22 m/s, 2.50 m/s, 2.78 m/s, 3.33 m/s, their self-reported preferred speed for a 5,000 m run, their preferred speed minus 0.14 m/s, and their preferred speed plus 0.14 m/s in a randomized order. At each speed, the participants ran for 150 s once the treadmill reached the target speed. An initial segment at the beginning of this trial was excluded for synchronization purposes. We selected a 120-s window following this segment for analysis. The final portion of the trial was omitted to avoid potential deceleration effects. Participants omitted speeds they felt uncomfortable running and did not repeat any speed. The resulting dataset comprised 242 trials across 43 participants.

Three measurement systems collected data simultaneously. First, the Xsens Link system (Xsens Technologies, Movella; Enschede, The Netherlands) recorded 3D acceleration and 4D quaternion data at 240 Hz using IMUs. These IMUs were attached by the researchers with straps, tape, a fitted T-shirt, and a belt over the L3–L5 spinal segments for the IMU at the pelvis. Although the system consisted of 17 IMUs, only the IMUs on the right foot and pelvis were used in this study. We selected these two IMUs because the foot-mounted sensor captures local kinematics at the distal segment, whereas the pelvis-mounted sensor provides information on whole-body motion as a proxy for center-of-mass movement. Second, an instrumented treadmill (Motek, Motek Medical B.V.; Houten, Netherlands) measured ground reaction forces at 1,200 Hz. Third, a 3D motion capture system (Vicon Motion Systems; Los Angeles, United States) tracked the position of 40 markers and four clusters of three markers at 120 Hz. Markers were placed according to the extended full-body plugin gait model (Vicon, 2024).

The force plates and 3D motion capture system collected data synchronously through Vicon Nexus. To synchronize these data sets with the IMU-collected data, we maximized the correlation of the acceleration signals of the IMU on the pelvis and the marker positioned on this IMU.

### 2.3 Musculoskeletal modeling

Musculoskeletal modeling was used to compute the SSL. The Gait2392 model served as the generic musculoskeletal model. A factor of ten was applied to the maximum isometric force of all muscles to prevent the activation profile of the soleus, medial gastrocnemius, and lateral gastrocnemius from reaching the upper limit of one, i.e., maximal activation. We scaled the generic model for every subject based on a static trial using OpenSim’s (Delp et al., 2007) scaling tool.

Next, inverse kinematics computed joint angles by fitting the subject’s model to the marker data. This was followed by inverse dynamics, combining kinematics and ground reaction forces, both filtered by a low-pass Butterworth filter with a cutoff frequency of 15 Hz. Subsequently, static optimization and joint reaction analysis estimated individual muscle forces and joint contact forces, respectively. The static optimization failed for 20 trials, due to muscle equilibrium failure, and hence these trials were excluded from the rest of the study.

### 2.4 Data processing

#### 2.4.1 IMU-collected data

The raw acceleration and quaternion signals collected by the IMUs were further processed without performing any post-hoc correction of the IMUs orientation. We computed the angular velocity ω of an IMU from the quaternion q at time t as $\omega \left(t\right)=\frac{2}{\Delta t}q{\left(t\right)}^{-1}q\left(t+\Delta t\right)$ with $q{\left(t\right)}^{-1}$ the conjugate of the quaternion q(t) . The acceleration signals were filtered by a fifth-order Butterworth filter with a 40 Hz cutoff frequency. Similar to the study by Bogaert et al. (2024), we identified initial contact (IC) and toe-off (TO) events based on the vertical acceleration signal crossing the thresholds of 0.18 g (with $g=9.81\;m/s^{2}$ ) and −0.25 g, respectively. Additional constraints ensured a decrease in acceleration before IC and TO, and an increase after IC. The −0.25 g threshold was gradually increased if no TO event was detected.

#### 2.4.2 Structure-specific load and data selection

The SSL of the Achilles and patellar tendons and the knee and ankle joint was calculated using musculoskeletal modelling.

• The Achilles tendon force was calculated as the sum of the forces of the soleus, medial gastrocnemius, and lateral gastrocnemius.

• The patellar tendon force was calculated as the sum of the forces of the rectus femoris, vastus medialis, vastus intermedius, and vastus lateralis.

• The knee contact force was calculated from the joint reaction analysis output as the three-dimensional resultant force of the knee joint reaction force components acting on the tibia.

• The ankle contact force was calculated from the joint reaction analysis output as the three-dimensional resultant force of the ankle joint reaction force components acting on the talus.

Because the SSL outputs were derived from musculoskeletal modeling with static optimization, which computes forces independently at each time point, a low-pass filter was applied. The calculated SSLs were filtered by a fifth-order Butterworth filter with a 20 Hz cutoff frequency and normalized to the participant’s body weight (BW). The SSL of a running trial was divided into individual steps based on the vGRF intersecting the 50 N threshold. This is in contrast to the gait events for the IMU data, which were estimated from the acceleration signals to reflect realistic deployment conditions where only IMUs are available. GRF-detected steps that could not be matched to a corresponding acceleration-detected step were excluded. Additionally, we omitted steps with a stance phase duration longer than 0.5 s or shorter than 0.15 s, a total step duration exceeding 0.6 s or below 0.21 s, or those showing signs of data corruption (e.g., signal dropout). Steps involving an SSL that fall outside the expected range were not included. For every step, the peak, impulse, and average loading rate of the SSL for the four musculoskeletal structures were calculated:

• Peak: The peak was calculated as the maximum of the SSL during the stance phase, expressed in units of BW.

• Impulse: The impulse was calculated as the integral of the SSL from initial contact until toe-off. The impulse obtained from its integration was expressed in units of BW ⋅ frame. This value was converted to BW ⋅ s by dividing by the effective sampling rate of the stance phase, computed as the number of frames in the time-normalized SSL-time curve (i.e., 100 frames) divided by the duration of the stance phase.

• Average loading rate: The average loading rate was computed as the average slope of the SSL–time curve over a defined interval. Specifically, the average loading rate was calculated as $average\;loading\;rate=\frac{SSL_{p_{\mathrm{upper}}}-SSL_{p_{\mathrm{lower}}}}{t_{p_{\mathrm{upper}}}-t_{p_{\mathrm{lower}}}}$ where $SSL_{p}$ is the force at p% of the interval of interest and $t_{p}$ the corresponding time point. For the Achilles tendon force, patellar tendon force, and ankle contact force, $p_{\mathrm{lower}}=20\%$ and $p_{\mathrm{upper}}=80\%$ of the interval between initial contact and peak force. For the knee contact force, $p_{\mathrm{lower}}=10\%$ and $p_{\mathrm{upper}}=40\%$ of the interval between initial contact and toe-off. The time difference $\left(t_{p_{\mathrm{upper}}}-t_{p_{\mathrm{lower}}}\right)$ was obtained from the difference in frame numbers and divided by the effective sampling rate of the time-normalized curve, yielding units of seconds. Loading rates are therefore expressed in BW/s.

We determined the step side of every step based on the relative position of the markers on the first metatarsal of the left and right foot. We included only right-side steps in the subsequent analyses to avoid the complexity of learning two distinct movement patterns within a single model, as the relationship between signals (e.g., medial-lateral acceleration and SSL) may differ between sides. This resulted in a total of 30,010 steps across the 43 subjects. All these processing steps were performed in Python 3.10.

### 2.5 Machine learning model

We trained a machine learning model to estimate the time-normalized (100 timestamps) SSL during the stance phase of a running step. The model has access to two types of data:

1. Raw time series measurements. It receives the individual measurement of each acceleration and angular velocity value at each time step. Hence, this yields a multivariate time series of shape 12×100 : each of the 2 sensors yields 3 acceleration signals and 3 angular velocity signals. All signals are time-normalized to have 100 values.
2. Tabular features. This includes:
  2.1. Subject-specific features. It considers four characteristics of the person (body mass, sex, age, and height) and two shoe characteristics (shoe length and sole thickness).
  2.2. Biomechanical features. First, we computed the stance duration and step duration. Second, we calculated the acceleration impulse of the stance phase from each of the six acceleration signals. Third, we computed the maximum, mean, root mean square, sample entropy, and skewness of each of the six acceleration signals. Finally, we computed the maximum and mean angular velocity from each of the six angular velocity signals. The last two categories of features were extracted using TSFresh (Christ et al., 2018). This yields 50 biomechanical features in total.

Time-series measurements were first passed through two bidirectional Long Short-Term Memory (LSTM) layers with 20 units each, capturing forward and backward temporal dependencies. The output of these layers was concatenated with the subject-specific and biomechanical features and subsequently batch-normalized to improve training stability. A dropout layer, with a dropout rate of 0.2, was applied before passing the data through a fully connected dense layer of 20 neurons with a rectified linear unit (ReLU) activation function. A second dropout layer with a 0.2 dropout rate preceded the final dense layer with a linear activation function, which estimated the SSL at each time step. This model will be denoted as the hybrid-LSTM model, as it takes both time series and tabular features as input. The peak, impulse, and average loading rate were computed from the estimated SSL during the stance phase.

To assess the relative value of the different types of features, we trained two additional models. First, the TS-LSTM model only considers the raw time series measurements. That is, the neural network is responsible for learning the right representation of the SSL from the time series. This model uses the same architecture as the hybrid-LSTM model, except it does not perform the catenation step after the bidirectional LSTM layers, as it does not use the subject-specific or biomechanical features. Second, the F-NN model only uses the subject-specific and biomechanical features. This model begins with a fully connected layer containing 20 neurons and a ReLU activation function. This is followed by a dropout layer with a dropout rate of 0.2, to help prevent overfitting. A second dense layer, with 20 neurons and ReLU activation, is then applied, followed by another dropout layer with a 0.2 dropout rate. The final layer is a fully connected dense layer with 100 neurons and a linear activation function.

To investigate how informative each IMU is on its own, we trained two variants of the hybrid-LSTM model: (1) only used data from the IMU on the right foot, and (2) only used data from the IMU on the pelvis. For both variants, time series and biomechanical features were restricted to those extracted from signals of the respective IMU, yielding time series with a dimension of 6×100 (three accelerometer and three gyroscope channels) and 26 biomechanical features (i.e., stance and step duration, six features per acceleration channel, and two features per angular velocity channel).

Model training and evaluation followed a five-fold cross-validation (CV) approach on the subject level (Davis et al., 2024). This ensures that all data for a single subject appears in the same fold to avoid information leakage. Within each fold, the training data is further subdivided using a subject-level split with five-sixths used for training and one-sixth as a validation set. Standardization was performed per fold, with scalers fitted on the training data. Models were trained using the Adam optimizer with an initial learning rate of 0.0001, which exponentially decayed by a factor of 0.90 every 10,000 steps. Training employed the mean squared error loss function and an early stopping criterion based on the validation loss, with a patience of 30 epochs. The maximum number of epochs was set to 500, and a batch size of 64 was used. Separate models were trained for each musculoskeletal structure (Achilles, patellar, knee, and ankle).

To provide a reference for model performance, two baseline models were implemented: (1) a linear regressor with L1 regularization (regularization strength tuned between 0.0001 and 100 based on validation loss), using tabular features, and (2) a mean regressor which computes the average time-normalized SSL from the training set (i.e., it makes a constant prediction.^1^ Both models were trained using the subject-level five-fold CV approach discussed above. The linear regressor models were trained with an Adam optimizer, and early stopping was determined based on validation loss. The mean regressor was trained without a separate validation set.

The mean square error (MSE), the mean absolute percentage error (MAPE), and the coefficient of determination ( ${\text{R}}^{2}$ ) assessed the model’s performance on the estimation of the SSL during the stance phase. From the estimated SSL during the stance phase, peak, impulse, and loading rate were calculated, as described in Section 2.4.2, and assessed using MSE and MAPE. Model training and evaluation were carried out in Python 3.9 using TensorFlow and scikit-learn.^2^

### 2.6 Model analyses

Stance-phase SSL estimation performance across models was evaluated using a mixed-effects model. For each tissue, we fitted a separate mixed-effects model with the MSE of the step as the dependent variable, ML model type as a categorical fixed effect (levels: dual-IMU hybrid-LSTM, dual-IMU TS-LSTM, dual-IMU F-NN, pelvis-IMU hybrid-LSTM, and foot-IMU hybrid-LSTM), and subject and step identification number as a random effect. The sample size for each mixed-effects model was 150,050. A Bonferroni correction was applied to correct for multiple comparisons across the four tissues.

Uncertainty in the estimated SSL values produced by the TS-LSTM models was quantified using Monte Carlo dropout (Gal and Ghahramani, 2016). On the test data, the dropout layers were retained and 100 forward passes were performed for each running step, yielding a distribution of values at each time point. The time-resolved uncertainty of the SSL during the stance phase was quantified as the standard deviation across the 100 estimates at each time stamp. To quantify the uncertainty of the peak SSL, impulse, and average loading rate, these derived characteristics were computed from the 100 forward passes for each running step and we reported the standard deviation of their respective values.

The statistical analysis was carried out in R 4.2.2 using lmerTest, and Monte Carlo dropout was carried out in Python 3.9 using Tensorflow and scikit-learn.

## 3 Results

### 3.1 Participants

Table 1 summarizes the descriptive characteristics of the 43 participants of this study. The age ranged from 18 to 49, and the running experience ranged from none to running more than 100 km per week.

**TABLE 1:** Summary of participant characteristics grouped by sex, reported as mean values ± standard deviation.

Characteristic	Male	Female
Count	36	7
Mass (kg)	79.8 ± 9.5	63.2 ± 2.9
Age (years)	24.4 ± 6.7	22.6 ± 3.5
Height (cm)	186.3 ± 7.4	173.1 ± 5.9
Shoe length (cm)	31.0 ± 1.2	28.4 ± 1.0
Sole thickness (cm)	3.3 ± 0.8	3.1 ± 1.2

### 3.2 SSL estimation performance

**FIGURE 1:**
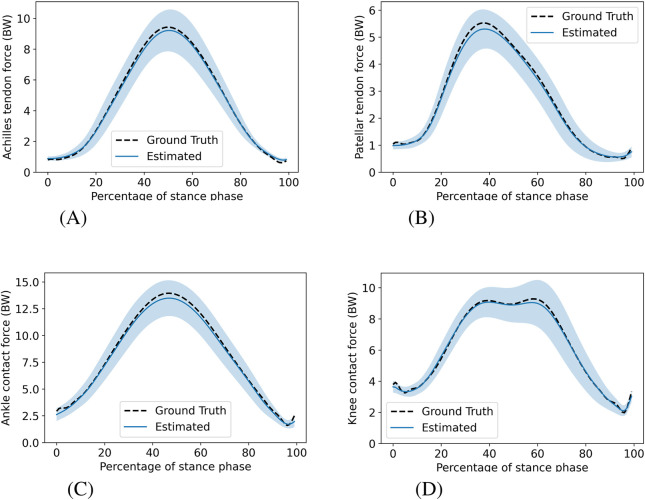
Mean SSL during the stance phase, averaged across all speeds for the hybrid-LSTM model using both IMUs and the ground truth. The shaded regions indicate ± one standard deviation for the hybrid-LSTM model. **(A)** Achilles tendon force, **(B)** Patellar tendon force, **(C)** Ankle contact force, **(D)** Knee contact force.

Figure 1 shows the mean SSL estimated by the hybrid-LSTM model using both IMUs, with corresponding standard deviations, alongside mean ground truth values during the stance phase for all four musculoskeletal structures. A similar comparison for three specific speeds (8 km/h, 10 km/h, and 12 km/h) is provided in the Supplementary Material (Supplementary Figure S1). Table 2 summarizes the performance of the hybrid-LSTM models using both IMUs, linear regression models, and mean regressors. The hybrid-LSTM models performed well across all structures, achieving the highest ${\text{R}}^{2}$ (0.93) for Achilles tendon force and the lowest MAPE (12.37%) for knee contact force. The hybrid-LSTM model achieved similar accuracy to linear regression models for Achilles, patellar, and ankle SSL (an increase in ${\text{R}}^{2}$ of 0.02 or less), but demonstrated superior performance for knee SSL (an increase in ${\text{R}}^{2}$ of 0.06). Across all structures, the hybrid-LSTM models consistently outperformed the mean regressors.

**TABLE 2:** The macro-averaged MSE, MAPE, and ${\text{R}}^{2}$ values for estimating the SSL for the Achilles tendon, patellar tendon, ankle, and knee using both IMUs for the hybrid-LSTM, linear regression, and mean regression models. The best-performing values are indicated in bold.

Structure	Model	MSE	MAPE	${\text{R}}^{2}$
Achilles	Hybrid-LSTM	**0.75**	17.89	**0.93**
Achilles	Linear regressor	0.89	**17.02**	0.92
Achilles	Mean regressor	1.22	20.49	0.87
Patellar	Hybrid-LSTM	**0.49**	**22.87**	**0.83**
Patellar	Linear regressor	0.71	25.67	0.81
Patellar	Mean regressor	0.56	23.73	0.79
Ankle	Hybrid-LSTM	1.93	14.59	0.89
Ankle	Linear regressor	**1.73**	**13.82**	**0.91**
Ankle	Mean regressor	2.38	15.39	0.86
Knee	Hybrid-LSTM	**0.96**	**12.37**	**0.86**
Knee	Linear regressor	1.51	14.93	0.80
Knee	Mean regressor	1.41	14.41	0.79

The mean ground truth values for SSL peak, impulse, and average loading rate are reported in the Supplementary Material (Supplementary Table S1). We derived SSL characteristics from the estimated stance-phase SSL profiles and summarized the performance of the hybrid-LSTM models using both IMUs, linear regression models, and mean regressors in Table 3.

**TABLE 3:** The macro-averaged MSE and MAPE values for estimating the SSL characteristics for the Achilles tendon, patellar tendon, ankle, and knee using both IMUs for the hybrid-LSTM, linear regression, and mean regression models. The best-performing values are indicated in bold.

Structure	Model	Peak		Impulse		Loading rate	
		MSE	MAPE	MSE	MAPE	MSE	MAPE
Achilles	Hybrid-LSTM	**1.31**	**9.02**	0.033	11.62	**233.6**	**12.18**
Achilles	Linear regressor	2.00	11.05	**0.032**	**10.99**	397.8	15.63
Achilles	Mean regressor	2.52	13.14	0.040	13.12	442.3	18.69
Patellar	Hybrid-LSTM	**1.02**	**14.15**	**0.018**	**15.04**	**333.9**	**23.91**
Patellar	Linear regressor	1.50	18.52	0.027	19.15	582.4	33.13
Patellar	Mean regressor	1.22	16.54	0.020	16.86	360.8	26.96
Ankle	Hybrid-LSTM	**2.82**	**9.16**	0.101	11.42	**515.8**	**14.76**
Ankle	Linear regressor	3.13	9.42	**0.075**	**10.30**	557.1	15.06
Ankle	Mean regressor	4.24	11.56	0.092	11.46	785.2	19.05
Knee	Hybrid-LSTM	**1.73**	**9.28**	**0.044**	**9.90**	**298.4**	**19.01**
Knee	Linear regressor	2.77	11.68	0.052	11.12	404.5	22.01
Knee	Mean regressor	2.78	11.74	0.049	10.78	394.0	23.27

**FIGURE 2:**
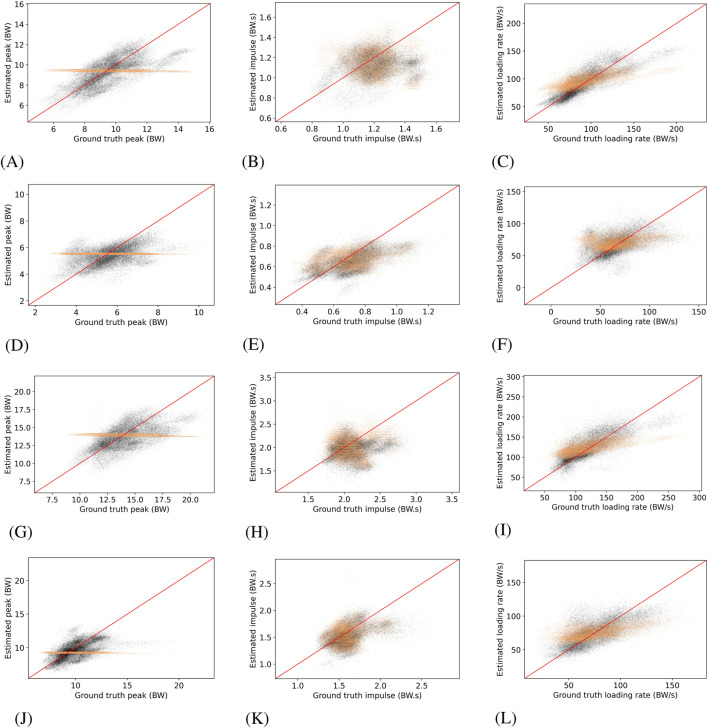
Estimated versus ground truth SSL characteristics for Achilles tendon, patellar tendon, ankle, and knee. Black dots show estimations from the hybrid-LSTM model using both IMUs, orange dots represent the mean regressor estimates, and the red line indicates perfect agreement between estimates and ground truth. **(A)** Achilles tendon force peak, **(B)** Achilles tendon force impulse, **(C)** Achilles tendon force loading rate, **(D)** Patellar tendon force peak, **(E)** Patellar tendon force impulse, **(F)** Patellar tendon force loading rate, **(G)** Ankle contact force peak, **(H)** Ankle contact force impulse, **(I)** Ankle contact force loading rate, **(J)** Knee contact force peak, **(K)** Knee contact force impulse, **(L)** Knee contact force loading rate.

Overall, the hybrid-LSTM model using both IMUs yielded lower MAPE and MSE values than the mean regressor across all musculoskeletal structures and SSL characteristics, consistent with its superior stance-phase estimation performance. The superior accuracy of the hybrid-LSTM model for SSL characteristics estimation is evident in Figure 2, which displays estimated versus ground truth values for the SSL characteristics for both models for Achilles tendon, patellar tendon, ankle contact, and knee contact force.

The linear regression models generally performed worse in terms of MSE and MAPE across musculoskeletal structures and SSL characteristics than the hybrid-LSTM model using both IMUs. One exception to this trend is that linear regression outperformed the dual-IMU hybrid-LSTM models for the impulse of the Achilles and ankle SSL.

### 3.3 Effect of different feature sets on SSL estimation

Table 4 summarizes the performance of the hybrid-LSTM, TS-LSTM, and F-NN models using both IMUs in estimating SSL during the stance phase. The hybrid-LSTM model and TS-LSTM models yielded comparable (a drop in ${\text{R}}^{2}$ of 0.01 or less) and strong SSL-estimation performance with ${\text{R}}^{2}$ values ranging from 0.83 for the patellar tendon force to 0.93 (hybrid-LSTM model) and 0.92 (TS-LSTM model) for Achilles tendon force estimation. The F-NN models, which relied solely on tabular features, achieved ${\text{R}}^{2}$ values between 0.81 and 0.92, indicating performance broadly comparable (a drop in ${\text{R}}^{2}$ of 0.02 or less) to the hybrid- and TS-LSTM models.

**TABLE 4:** The macro-averaged MSE, MAPE, and ${\text{R}}^{2}$ values for estimating the SSL for the Achilles tendon, patellar tendon, ankle, and knee using both IMUs for the hybrid-LSTM, TS-LSTM, and F-NN models. The best-performing values are indicated in bold.

Structure	Model	MSE	MAPE	${\text{R}}^{2}$
Achilles	Hybrid-LSTM	**0.75**	17.89	**0.93**
Achilles	TS-LSTM	0.83	17.69	0.92
Achilles	F-NN	0.81	**16.73**	0.92
Patellar	Hybrid-LSTM	**0.49**	**22.87**	**0.83**
Patellar	TS-LSTM	0.54	24.97	**0.83**
Patellar	F-NN	0.51	23.17	0.81
Ankle	Hybrid-LSTM	1.93	14.59	0.89
Ankle	TS-LSTM	1.84	13.58	0.89
Ankle	F-NN	**1.77**	**12.58**	**0.90**
Knee	Hybrid-LSTM	**0.96**	**12.37**	**0.86**
Knee	TS-LSTM	1.06	13.16	0.85
Knee	F-NN	1.16	12.51	0.84

Table 5 presents the performance of the hybrid-LSTM, TS-LSTM, and F-NN models using both IMUs in estimating SSL peak, impulse, and average loading rate. The TS-LSTM models yielded overall comparable performance (an increase in MAPE of 0.52 or less, except an increase in MAPE of 2.01 for Achilles SSL loading rate) to the hybrid-LSTM model. The F-NN models showed slightly lower accuracy (increase in MAPE up to 3.41) compared to the hybrid-LSTM model but still outperformed the linear and mean regressors across all musculoskeletal structures and SSL characteristics, except for the impulse of Achilles and ankle SSL (see Table 3).

**TABLE 5:** The macro-averaged MSE and MAPE values for estimating the SSL characteristics for the Achilles tendon, patellar tendon, ankle, and knee using both IMUs for the hybrid-LSTM, TS-LSTM, and F-NN models. The best-performing values are indicated in bold.

Structure	Model	Peak		Impulse		Loading rate	
		MSE	MAPE	MSE	MAPE	MSE	MAPE
Achilles	Hybrid-LSTM	**1.31**	**9.02**	0.033	11.62	**233.6**	**12.18**
Achilles	TS-LSTM	1.46	9.54	0.036	12.14	337.5	14.19
Achilles	F-NN	1.75	12.43	**0.029**	**10.47**	331.3	15.19
Patellar	Hybrid-LSTM	1.02	14.15	**0.018**	**15.04**	333.9	23.91
Patellar	TS-LSTM	1.02	**13.50**	0.019	15.30	**323.7**	**22.18**
Patellar	F-NN	**0.98**	14.88	0.021	16.36	341.4	25.24
Ankle	Hybrid-LSTM	**2.82**	9.16	0.101	11.42	**515.8**	**14.76**
Ankle	TS-LSTM	**2.82**	**8.90**	0.095	11.07	527.3	15.07
Ankle	F-NN	3.43	9.72	**0.085**	**10.19**	634.8	16.39
Knee	Hybrid-LSTM	1.73	9.28	0.044	9.90	**298.4**	19.01
Knee	TS-LSTM	**1.57**	**8.60**	**0.033**	**8.63**	331.9	**18.87**
Knee	F-NN	1.93	9.06	0.048	9.33	316.1	20.00

### 3.4 Effect of using a single IMU for SSL estimation

Table 6 summarizes the performance of the dual-IMU hybrid-LSTM, pelvis-IMU hybrid-LSTM, and foot-IMU hybrid-LSTM models in estimating the SSL during the stance phase. The pelvis-IMU hybrid-LSTM models achieved ${\text{R}}^{2}$ scores comparable to the dual-IMU configuration (a drop in ${\text{R}}^{2}$ of 0.02 or less). In contrast, foot-IMU hybrid-LSTM models showed reduced performance for estimating knee and Achilles SSL (a drop in ${\text{R}}^{2}$ of 0.04 or more), while maintaining similar accuracy for patellar and ankle SSL (a drop in ${\text{R}}^{2}$ of 0.02 or less) compared to the dual-IMU counterpart.

**TABLE 6:** The macro-averaged MSE, MAPE, and ${\text{R}}^{2}$ values for estimating the SSL for the Achilles tendon, patellar tendon, ankle, and knee using both or a single IMU for the hybrid-LSTM models. The best-performing values are indicated in bold.

Structure	IMUs	MSE	MAPE	${\text{R}}^{2}$
Achilles	Pelvis and foot	**0.75**	17.89	**0.93**
Achilles	Pelvis	1.02	**17.80**	0.91
Achilles	Foot	1.11	19.11	0.89
Patellar	Pelvis and foot	**0.49**	22.87	0.83
Patellar	Pelvis	0.50	**22.05**	**0.84**
Patellar	Foot	0.59	24.19	0.81
Ankle	Pelvis and foot	1.93	14.59	**0.89**
Ankle	Pelvis	**1.87**	**13.68**	**0.89**
Ankle	Foot	2.06	14.40	0.88
Knee	Pelvis and foot	**0.96**	**12.37**	**0.86**
Knee	Pelvis	1.08	12.71	0.85
Knee	Foot	1.42	14.19	0.81

Table 7 presents the performance of the single- and dual-IMU hybrid-LSTM models on estimating SSL peak, impulse, and average loading rate. The performance of the hybrid-LSTM models declined slightly when utilizing a single IMU compared to both IMUs, with the pelvis-IMU model generally outperforming the foot-IMU counterpart. For example, in estimating the peak Achilles tendon force, the hybrid-LSTM model using both IMUs achieved an MAPE of 9.02%, compared to 11.20% and 12.53% for the pelvis- and foot-IMU models, respectively.

**TABLE 7:** The macro-averaged MSE and MAPE values for estimating the SSL characteristics for the Achilles tendon, patellar tendon, ankle, and knee using both or a single IMU for the hybrid-LSTM models. The best-performing values are indicated in bold.

Structure	IMUs	Peak		Impulse		Loading rate	
		MSE	MAPE	MSE	MAPE	MSE	MAPE
Achilles	Pelvis and foot	**1.31**	**9.02**	**0.033**	**11.62**	**233.6**	**12.18**
Achilles	Pelvis	2.24	11.20	0.044	12.96	411.1	15.53
Achilles	Foot	2.28	12.53	0.051	14.33	375.1	17.40
Patellar	Pelvis and foot	**1.02**	**14.15**	**0.018**	**15.04**	**333.9**	**23.91**
Patellar	Pelvis	1.03	14.29	0.021	15.76	343.0	24.03
Patellar	Foot	1.42	15.56	0.020	15.65	415.3	26.41
Ankle	Pelvis and foot	**2.82**	**9.16**	**0.101**	**11.42**	**515.8**	**14.76**
Ankle	Pelvis	3.40	10.29	0.109	12.12	678.0	16.10
Ankle	Foot	3.60	10.33	0.112	12.02	580.6	15.79
Knee	Pelvis and foot	**1.73**	9.28	**0.044**	9.90	**298.4**	**19.01**
Knee	Pelvis	1.89	**9.19**	0.045	**9.68**	380.5	20.35
Knee	Foot	2.46	11.06	0.059	11.33	386.3	21.25

The mixed-effects analysis revealed statistically significant differences among model types (p<0.001) , with the dual-IMU hybrid-LSTM specified as the reference category. In the remainder of the manuscript, we focus on reporting differences in performance metrics (e.g., R^2^ or MAPE) between models to support a practically oriented comparison of model performance.

## 4 Discussion

Accurate estimation of SSL during running is essential for understanding and preventing RROIs. This study demonstrates that SSL can be estimated during running across key RROI-prone structures using only one or two physical IMUs. Our machine learning models estimated both continuous SSL profiles during the stance phase and discrete SSL characteristics. These findings highlight the potential for a practical estimation of SSL during running.

### 4.1 SSL estimation performance

#### 4.1.1 Estimation of SSL during stance phase

The hybrid-LSTM models using both IMUs achieved the best performance for estimating the Achilles and patellar tendon force, and ankle and knee contact force throughout the stance phase. Although the simpler linear regression models performed comparably, in terms of ${\text{R}}^{2}$ , to the hybrid-LSTM models for Achilles, patellar, and ankle SSL, they offered worse performance when estimating knee SSL. As these simpler models offer benefits for embedding SSL estimation in wearable devices, combining low memory demands with fast processing capabilities, they may be sufficient for some, but not all, musculoskeletal structures.

Our results are comparable with previous studies despite the fact that these studies used more complex measures, such as marker-based joint angles or simulated IMU signals as input. For instance, Liew et al. (2023) estimated the SSL with a weighted average correlation of 0.97 and 0.98 for ankle and knee contact forces, similar to our hybrid-LSTM models’ correlation of 0.98 and 0.96. However, the reliance on joint angles complicates data acquisition and limits real-world applicability. Another study (David et al., 2024), estimating SSL using four simulated IMUs, reported higher accuracy for patellar and ankle SSL ( ${\text{R}}^{2}$ of 0.92 and 0.93, respectively), compared to our hybrid-LSTM model using both IMUs ( ${\text{R}}^{2}$ of 0.83 and 0.89, respectively). However, the performance of their four-IMU model remained similar in predicting Achilles tendon force ( ${\text{R}}^{2}$ of 0.93) and knee contact force ( ${\text{R}}^{2}$ of 0.87 for their model and 0.86 for our hybrid-LSTM model). The use of simulated IMUs in these previous studies does not fully capture sensor noise and variability, which may affect model performance when transitioning to physical IMUs. In addition, their approach required twice as many IMUs, potentially increasing the complexity and burden of practical implementation. It is also important to note that variation in datasets across studies complicates performance comparisons, and thus, these literature benchmarks should be interpreted with caution.

#### 4.1.2 Estimation of SSL characteristics

Peak and loading rate were estimated most accurately by the hybrid-LSTM models, while impulse proved more challenging. This was evident both quantitatively, where improvements in impulse estimation over the mean regressor were marginal, and visually, as shown in Figure 2. As cumulative tissue damage scales with the number of load cycles and the load magnitude raised to a structure-specific power (Edwards, 2018), the performance of estimating peak SSL is crucial and underscores the potential practical applications of this approach.

Based on a previous study (Kwon et al., 2023) investigating walking with an immobilization boot, we used criteria for agreement categories for Achilles tendon force. To account for higher peak forces during running, inherently resulting in lower MAPE values when the absolute errors remain similar, we adopted 10% for good agreement and 15% for acceptable agreement. Using these criteria, the hybrid-LSTM models using both IMUs achieved good agreement for the peak SSL of the Achilles tendon, ankle joint, and knee joint, and acceptable agreement for the patellar tendon.

Compared to a study by Brund et al. (2021), who employed a mixed-effects regression model using anthropometric and sportwatch-derived variables, our hybrid-LSTM models using both IMUs achieved lower normalized mean absolute errors (calculated from proportion of prediction error) for peak and impulse Achilles and patellar tendon forces. They reported normalized mean absolute errors of 13% and 19% for peak Achilles and patellar tendon force, and 14% and 25% for impulse Achilles and patellar tendon force compared to our 9.2% and 13.8% for peak, and 12.0% and 15.1% for impulse, for the Achilles and patellar tendon force, respectively. While Brund et al.‘s model relied on more accessible input data, our results highlight the added value of wearable IMUs for improving estimation accuracy.

Our hybrid-LSTM model using both IMUs reached similar accuracy as the results of Rasmussen et al. (2023), using a machine learning approach relying on two simulated IMUs to estimate peak SSL. They reported normalized RMSEs of 0.18 for the Achilles and 0.12 for the patellar tendon comparable to our normalized RMSEs of 0.12 and 0.18, respectively. Our results highlight the feasibility of physical-IMU-based SSL estimation, taking a step towards translating SSL estimation models into real-world settings.

We observed higher errors when estimating the SSL impulse compared to the SSL peak or loading rate. One possible reason is that impulse is computed by integrating over time, and small errors made at each time step accumulate. Future work could explore strategies to improve estimating the SSL impulse, such as including temporal derivatives as input to the model, developing an end-to-end model that directly predicts impulse as opposed to deriving it from the SSL estimates for each time point during the stance phase, or including an additional term in the loss function that penalizes errors in estimated impulse to guide the model toward more accurate impulse SSL estimations.

Practical utility was assessed by determining whether the machine learning models can detect duty-factor–related changes in SSL. With increasing duty factor, peak Achilles tendon force, ankle contact force, and knee contact force decreased by 1.30 BW, 2.22 BW, and 1.73 BW, respectively (Bonnaerens et al., 2022). These changes exceed the square root of the MSE of the dual-IMU hybrid-LSTM model for peak Achilles, ankle, and knee SSL estimation (1.14 BW, 1.67 BW, and 1.31 BW, respectively), indicating that the model is likely capable of detecting meaningful changes in Achilles, ankle, and knee SSL associated with variations in duty factor, and supporting its potential practical utility. In contrast, the peak patellar tendon force decreased by approximately 0.59 BW with increasing duty factor (Bonnaerens et al., 2022). This value is below the square root of the MSE of the dual-IMU hybrid-LSTM model for patellar tendon SSL estimation (1.01 BW), suggesting that additional model improvements may be required to reliably capture variations in peak patellar tendon force due to changes in duty factor.

#### 4.1.3 Estimation of SSL: Overall

Overall, the results demonstrate the potential of the hybrid-LSTM models using both IMUs for estimating SSL continuously during the stance phase and the derived SSL characteristics, namely peak and average loading rate. However, further work is needed to improve the accuracy of the impulse of the estimated SSL.

The hybrid-LSTM models outperformed both baseline approaches, i.e., the mean and linear regressors. While the linear regression models achieved comparable performance on stance-phase SSL estimation for some musculoskeletal structures, they underperformed regarding the SSL characteristics. Given the relevance of SSL characteristics, particularly peak, in tissue damage and injury risk, these findings underscore the added value of hybrid-LSTM models over the linear regressors for SSL estimation.

Variability in sensor alignment across participants may be present in our dataset due to soft-tissue artifacts, belt movement, or individual IMU mounting differences. However, by performing subject-level cross-validation, our methodology setup tests the ability to generalize across the natural variation in IMU orientation and positioning.

### 4.2 Effect of different feature sets on SSL estimation

The TS-LSTM models, relying exclusively on time-series features, matched the hybrid-LSTM models’ performance in estimating stance-phase SSL and SSL characteristics. This finding indicates that TS-LSTM models can deliver similar accuracy without requiring the collection or computation of tabular features, thereby simplifying data acquisition and preprocessing. The F-NN models, which used only tabular features, performed comparably to hybrid-LSTM models for stance-phase SSL estimation and were similar or slightly inferior for SSL characteristics. The marginal performance advantage of TS-LSTM over F-NN model in SSL characteristic estimation suggests that time-series data captures information not fully represented by biomechanical or subject-specific features. Given the importance of SSL characteristics, such as peak SSL, F-NN models offer less practical value relative to the TS-LSTM models.

### 4.3 Effect of using a single IMU for SSL estimation

The pelvis-IMU hybrid-LSTM model outperformed the foot-IMU model in estimating patellar and knee SSL during the stance phase as well as for the SLL characteristics, while both performed similarly for the other musculoskeletal structures. When compared with the dual-IMU configuration (pelvis and foot), the single pelvis-IMU hybrid-LSTM model achieved comparable ${\text{R}}^{2}$ values for stance-phase SSL estimation (a drop in ${\text{R}}^{2}$ of 0.02 or less) and exhibited similar or only marginal reductions in accuracy for the SSL characteristics (an increase in MAPE of 1.34 or less, except for Achilles SSL peak and loading rate where the drop is 2.18 and 3.35, respectively). These findings suggest that the additional foot IMU provides limited accuracy gains relative to the increased setup complexity. Supplementary Material (Supplementary Table S2, 3) show the performance of the dual- and single-IMU TS-LSTM models, and indicate that SSL estimation during running can be achieved reliably using time series data from a single pelvis-mounted IMU, reducing the computational complexity. The overall increased practicality of the pelvis-IMU TS-LSTM model over the dual-IMU hybrid-LSTM model could make SSL estimation more accessible, potentially encouraging broader adoption among runners.

Supplementary Figure S2 and Supplementary Table S6 in the Supplementary Material report the uncertainty associated with each SSL indicator when using pelvis-IMU TS-LSTM models. Across tissues, uncertainty was lowest at the beginning and end of the stance phase and increased near the mid-stance phase. Specifically, higher uncertainty was observed around 50% of the stance phase for the Achilles tendon, ankle contact, and knee contact forces, and around 35% of the stance phase for the patellar tendon forces. These patterns reflect the temporal evolution of the estimated SSL during the stance phase, which generally attain their highest values near mid-stance phase and decrease toward initial contact and toe-off. This suggests that model uncertainty increases when the estimated SSL values are higher.

To quantify the contribution of each IMU signal to the pelvis-IMU TS-LSTM models, we performed a signal ablation analysis in which model performance was re-evaluated after systematically omitting each IMU signal (Supplementary Material, Supplementary Tables S4, S5). Leaving out any one of the six IMU signals (three acceleration and three angular velocity signals) showed that the pelvis-IMU TS-LSTM models used information redundantly across channels: in most cases, removing a single signal caused only modest changes in model performance. However, structure-specific sensitivities emerged in terms of the effect of excluding particular signals. Achilles tendon force estimates were most affected by removing the angular velocity about the medial–lateral axis, whereas patellar tendon force estimates were most impacted by omitting the anterior–posterior acceleration. Ankle and knee force estimates were more sensitive to the removal of, respectively, vertical acceleration and angular velocity about the vertical axis. Thus, despite redundancy across signals, some axes still carry unique, valuable information for estimating SSL. Notably, the axes correspond to the sensor’s local coordinate frame and therefore only approximately align with anatomical anterior–posterior, medial–lateral, and vertical directions.

### 4.4 Limitations

This study has several limitations that should be considered when interpreting the findings. The analysis focused exclusively on right-side steps. Although a left-side counterpart is expected to perform similarly, extending the dual-IMU hybrid-LSTM model to SSL estimation of left-side steps would likely require an IMU on the left foot. Nevertheless, the pelvis-IMU model, which achieved comparable performance for SSL estimation of right-side steps, avoids this requirement.

Although the presented method shows promise for real-world use, it was developed and evaluated in a laboratory setting. Therefore, the generalizability of the SSL estimation models from controlled laboratory conditions to real-world outdoor contexts remains uncertain. Factors such as terrain irregularities, slopes, footwear, and weather conditions (Ahamed et al., 2018) may alter running patterns and potentially affect model performance. The exclusive reliance on treadmill running further limits ecological validity. In addition, the running speeds in this study were limited to the range of 7 km/h to 14 km/h, which can further limit the generalizability, as running speed can influence the relationship between IMU signals and SSL. Transfer learning strategies may offer a potential avenue to improve model generalizability by adapting pretrained models to new running conditions using limited additional data (Zhang L. et al., 2022).

Finally, the generalizability of the SSL estimation models is constrained by the demographic composition of the dataset of this study, which predominantly consisted of young male adults. Previous research has shown sex-related differences in SSL surrogates during running. Female runners exhibit higher peak knee joint reaction forces (Rubio et al., 2023), peak patellar tendon loading surrogate (Sinclair and Taylor, 2015), and lower peak Achilles tendon loading surrogate (Greenhalgh and Sinclair, 2014). Although these are not direct SSL measures, they may still indicate sex-specific SSL patterns. In addition, age and obesity have been shown to influence ground reaction forces (Kline and Williams, 2015; Vincent et al., 2020), which may imply age- and body-mass-index-related differences in SSL. The limited variability in these characteristics in our dataset, therefore, restricts the extent to which the findings can be generalized to more diverse populations.

### 4.5 Future work

Several directions for future research emerge from this work. In line with common practice in IMU-based running biomechanics, this study adopted a two-sensor configuration, with IMUs placed on the pelvis and right foot, locations that are among the most frequently used in the literature (Xiang et al., 2022). While this setup offers a practical setup, additional or alternative IMU placements (e.g., on the shank or thigh) may provide complementary information relevant for SSL estimation. Future work should therefore investigate optimal IMU placement strategies that balance estimation accuracy with practicality in real-world settings.

The IMU-based models presented here offer a practical alternative to traditional musculoskeletal modeling for estimating SSL. While these models could potentially support near-real-time SSL estimation, temporal performance was not explicitly evaluated in this study. The pelvis-IMU TS-LSTM models with associated scaler required 270,149 bytes per tissue and 0.0485 s (standard deviation of 0.0127 s for 1,000 iterations per tissue; conducted on laptop CPU, Intel Core i7-11800H, 32 GB RAM, Windows 11) to estimate SSL during stance from features for a single running step and tissue. Future work should assess the latency, accuracy, and computational requirements of near-real-time SSL estimation in real-world settings, including the implementation of the models and necessary data processing on edge devices.

This study used musculoskeletal modeling to derive ground-truth SSL values during running. Although musculoskeletal modeling is a well-established approach for estimating SSL, it relies on multiple assumptions and simplifications that can affect the resulting simulations and introduce inherent estimation errors. Because the machine learning models were trained to reproduce musculoskeletal-derived outputs, future work should investigate how variations in the underlying musculoskeletal simulations influence the learned SSL estimates. Such analyses could be performed through parameter sensitivity studies (e.g., tendon slack length, tendon stiffness, muscle force scaling) to better understand the impact of modeling assumptions on simulated SSL. In addition, future studies could explore the effects of individualizing musculoskeletal model parameters on estimated SSL.

Lastly, this study focused on the SSL of four tissues susceptible to running-related overuse injuries: the Achilles tendon, patellar tendon, ankle, and knee. However, other tissues are also susceptible to running-related overuse injuries. Further research is needed to extend the SSL estimation to additional tissues.

## 5 Conclusion

In conclusion, this study demonstrated that machine learning models can estimate SSL while running using data from one or two IMUs combined with subject-specific features. The models captured the temporal evolution of SSL during the stance phase and the derived SSL characteristics, namely peak SSL and average loading rate, across multiple musculoskeletal structures at risk for RROIs. The TS-LSTM models matched the hybrid-LSTM models’ accuracy, making them a practical choice for SSL estimation. Importantly, a single pelvis-mounted IMU combined with the TS-LSTM approach achieved performance comparable to the dual-IMU hybrid-LSTM configuration. This simplified setup, which avoids both the need for a second IMU and additional tabular features, represents the most practical and scalable option for real-world applications. Collectively, the findings of this study highlight the potential of this approach for SSL monitoring in natural running environments.

## Data Availability

The raw data supporting the conclusions of this article will be made available by the authors, without undue reservation.

## Footnotes

1 This model assumes there is no relationship between the features and the target variable

2 The implementation is available on https://github.com/ML-KULeuven/StructureSpecificLoad-LSTM.
