# Supplementary material for: Machine learning-based estimation of structure-specific load around the ankle and knee joint during running using IMU data
Source: Front Bioeng Biotechnol. 2026 Feb 11;14:1710980. doi: 10.3389/fbioe.2026.1710980 (PMC12932605; doi:10.3389/fbioe.2026.1710980)
Supplement: Supplementary file 1 [file Supplementaryfile1.pdf]

Supplementary Material

1 SUPPLEMENTARY FIGURES

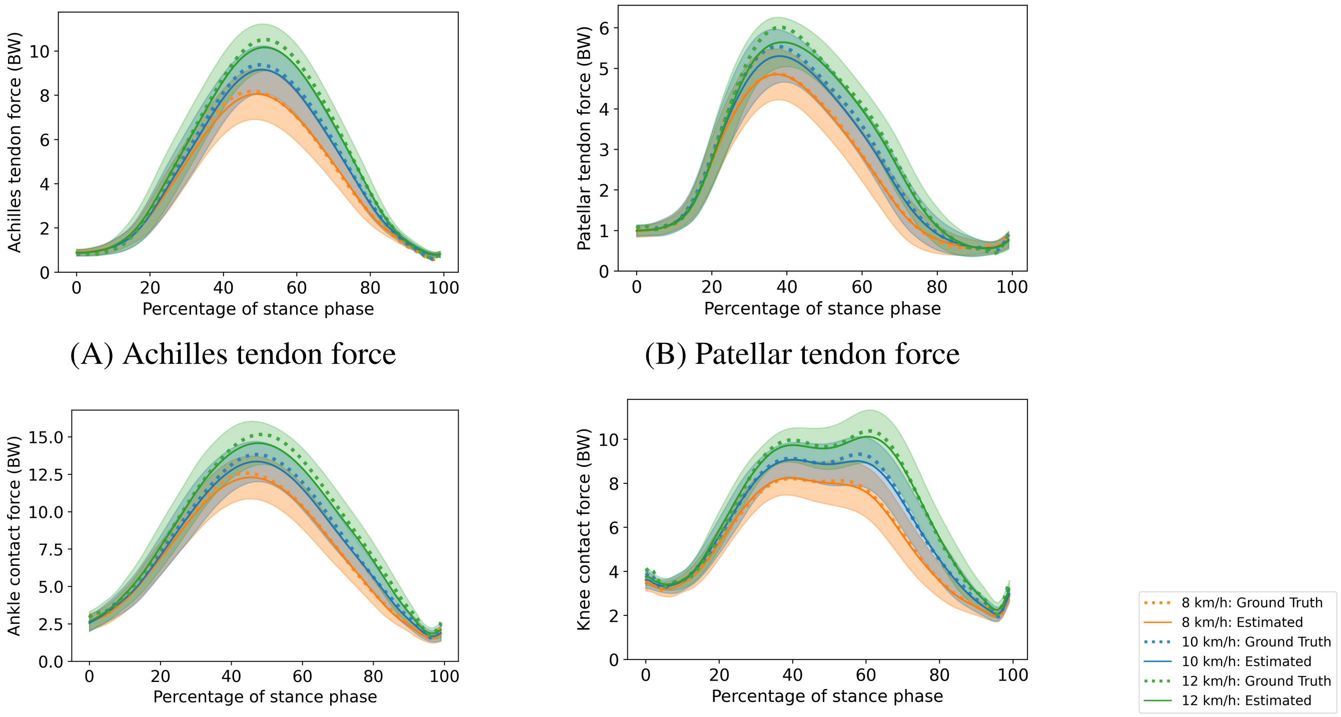

(A) Achilles tendon force (B) Patellar tendon force

(C) Ankle contact force (D) Knee contact force (E) Legend

Figure S1. Mean SSL during the stance phase at three running speeds (8, 10, and 12 km/h). Solid lines with shaded areas show the estimations by the hybrid-LSTM model using both IMUs (mean $\pm$ standard deviation), and dotted lines indicate the mean ground truth.

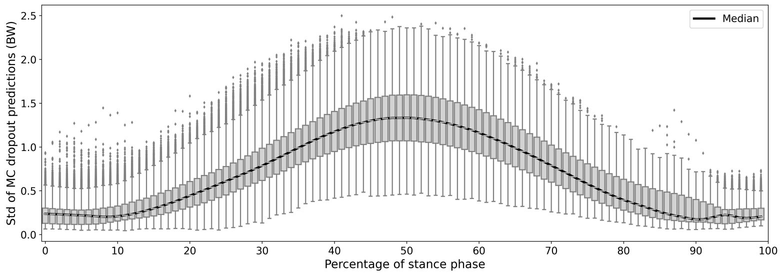

(A) Achilles tendon force

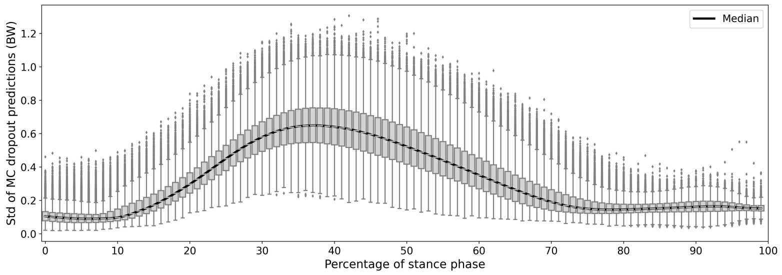

(B) Patellar tendon force

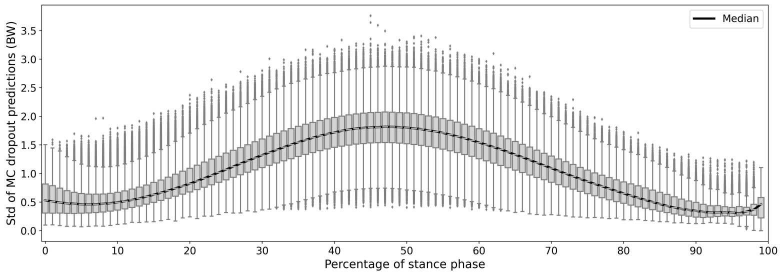

(C) Ankle contact force

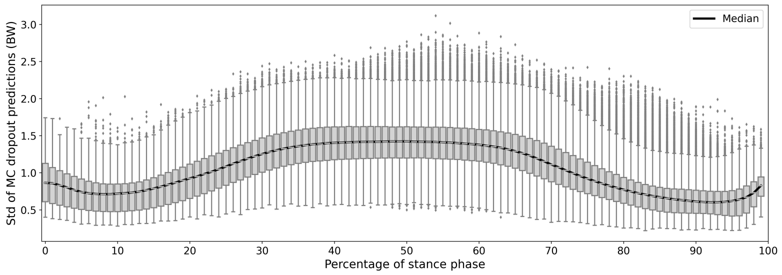

(D) Knee contact force

Figure S2. Standard deviation (std) across the 100 Monte Carlo dropout estimations of SSL during the stance phase for Achilles tendon force, patellar tendon force, ankle contact force, and knee contact force. Boxplots represent the distribution of the standard deviations across all steps for each of the 100 time points of the stance phase. The black line overlays the median across all time points.

2 SUPPLEMENTARY TABLES

Table S1. The mean ± standard deviation of the peak, impulse, and average loading rate across the data set of the ground truth values for the Achilles tendon force, patellar tendon force, ankle contact force, and knee contact force.

Structure	Peak (BW)	Impulse (BW·s)	Loading rate (BW/s)
Achilles	9.54 ± 1.57	1.20 ± 0.13	93.88 ± 26.94
Patellar	5.63 ± 1.10	0.71 ± 0.15	66.20 ± 18.96
Ankle	14.10 ± 2.01	2.12 ± 0.21	121.87 ± 35.18
Knee	10.00 ± 1.50	1.61 ± 0.18	75.13 ± 22.88

Table S2. The macro-averaged MSE, MAPE, and R^2 values for estimating the SSL for the Achilles tendon, patellar tendon, ankle, and knee using both or a single IMU for the TS-LSTM models. The best-performing values are indicated in bold.

Structure	IMUs	MSE	MAPE	R^2
Achilles	Pelvis and foot	**0.83**	**17.69**	**0.92**
Achilles	Pelvis	1.07	19.30	0.91
Achilles	Foot	1.09	19.23	0.89
Patellar	Pelvis and foot	0.54	24.97	0.83
Patellar	Pelvis	**0.50**	**22.18**	**0.84**
Patellar	Foot	0.65	25.87	0.80
Ankle	Pelvis and foot	**1.84**	**13.58**	**0.89**
Ankle	Pelvis	1.90	13.98	**0.89**
Ankle	Foot	2.81	17.19	0.84
Knee	Pelvis and foot	**0.96**	**12.37**	**0.86**
Knee	Pelvis	1.05	12.72	**0.86**
Knee	Foot	1.51	15.02	0.79

Table S3. The macro-averaged MSE and MAPE values for estimating the SSL characteristics for the Achilles tendon, patellar tendon, ankle, and knee using both or a single IMU for the TS-LSTM models. The best-performing values are indicated in bold.

		Peak		Impulse		Loading rate	
Structure	IMUs	MSE	MAPE	MSE	MAPE	MSE	MAPE
Achilles	Pelvis and foot	**1.46**	**9.54**	**0.036**	**12.14**	**337.5**	**14.19**
Achilles	Pelvis	2.24	11.35	0.048	13.68	367.8	15.52
Achilles	Foot	2.17	12.43	0.049	14.15	355.5	15.98
Patellar	Pelvis and foot	**1.02**	**13.50**	**0.019**	**15.30**	**323.7**	**22.18**
Patellar	Pelvis	1.04	14.39	0.021	15.66	346.3	24.15
Patellar	Foot	1.28	15.21	0.023	16.74	377.9	24.01
Ankle	Pelvis and foot	**2.82**	**8.90**	**0.095**	**11.07**	527.3	15.07
Ankle	Pelvis	3.30	10.31	0.097	11.56	680.6	16.49
Ankle	Foot	4.01	10.83	0.128	13.12	**510.8**	**15.00**
Knee	Pelvis and foot	**1.57**	**8.60**	**0.033**	**8.63**	331.9	**18.87**
Knee	Pelvis	1.96	9.77	0.046	9.98	**316.6**	19.00
Knee	Foot	2.55	11.16	0.058	11.25	396.6	20.56

Table S4. The macro-averaged MSE, MAPE, and R^2 values for estimating the SSL for the Achilles tendon, patellar tendon, ankle, and knee using the pelvis IMU for the TS-LSTM models, dropping different signals. Angular velocities are rotations about the listed axis. The best-performing values are indicated in bold. *Abbreviations:* Acceleration (Acc), angular velocity (Ang Vel), anterior-posterior (AP), medial-lateral (ML), and vertical (VT).

Structure	Dropped signal	MSE	MAPE	R^2
Achilles	None	1.07	19.30	0.91
Achilles	Acc AP	1.01	19.08	0.91
Achilles	Acc ML	1.03	18.73	0.91
Achilles	Acc VT	0.99	18.97	0.91
Achilles	Ang Vel AP	0.97	18.82	0.91
Achilles	Ang Vel ML	1.22	19.47	0.89
Achilles	Ang Vel VT	**0.84**	**17.47**	**0.92**
Patellar	None	0.50	22.18	0.84
Patellar	Acc AP	0.51	23.21	0.82
Patellar	Acc ML	**0.43**	**20.58**	**0.86**
Patellar	Acc VT	0.48	21.91	0.85
Patellar	Ang Vel AP	0.50	22.32	0.84
Patellar	Ang Vel ML	0.47	21.97	0.84
Patellar	Ang Vel VT	0.50	21.93	0.83
Ankle	None	1.90	13.98	0.89
Ankle	Acc AP	**1.65**	**13.32**	**0.91**
Ankle	Acc ML	1.91	13.82	0.89
Ankle	Acc VT	2.09	14.28	0.88
Ankle	Ang Vel AP	1.67	13.53	0.90
Ankle	Ang Vel ML	1.95	13.80	0.89
Ankle	Ang Vel VT	2.26	15.11	0.86
Knee	Pelvis	1.05	12.72	**0.86**
Knee	Acc AP	1.06	12.76	0.85
Knee	Acc ML	1.06	12.36	**0.86**
Knee	Acc VT	1.19	13.39	0.84
Knee	Ang Vel AP	**1.01**	**12.37**	**0.86**
Knee	Ang Vel ML	1.12	13.14	0.85
Knee	Ang Vel VT	1.09	12.83	0.85

Table S5. The macro-averaged MSE and MAPE values for estimating the SSL characteristics for the Achilles tendon, patellar tendon, ankle, and knee using the pelvis IMU for the TS-LSTM models, dropping different signals. Angular velocities are rotations about the listed axis. The best-performing values are indicated in bold. *Abbreviations:* Acceleration (Acc), angular velocity (Ang Vel), anterior-posterior (AP), medial-lateral (ML), and vertical (VT).

		Peak		Impulse		Loading rate	
**Structure**	**Dropped signal**	**MSE**	**MAPE**	**MSE**	**MAPE**	**MSE**	**MAPE**
Achilles	None	2.24	11.35	0.048	13.68	367.8	**15.52**
Achilles	Acc AP	1.78	10.39	0.038	**11.88**	432.1	16.73
Achilles	Acc ML	1.91	11.03	0.043	13.44	387.5	15.92
Achilles	Acc VT	2.02	11.35	**0.036**	12.41	410.1	16.79
Achilles	Ang Vel AP	1.70	**10.16**	0.040	12.64	**359.7**	16.13
Achilles	Ang Vel ML	2.71	13.05	0.051	14.43	394.8	15.97
Achilles	Ang Vel VT	**1.66**	10.24	0.037	12.03	381.8	15.77
Patellar	None	1.04	14.39	0.021	15.66	346.3	24.15
Patellar	Acc AP	1.04	14.23	0.020	15.71	**333.6**	**23.30**
Patellar	Acc ML	**0.91**	**13.19**	**0.018**	**13.75**	338.1	24.73
Patellar	Acc VT	1.08	14.77	0.021	15.25	339.5	24.91
Patellar	Ang Vel AP	1.10	14.91	0.021	15.67	339.7	24.77
Patellar	Ang Vel ML	1.05	14.33	0.019	15.10	340.8	24.46
Patellar	Ang Vel VT	1.04	14.78	0.022	16.69	302.4	23.52
Ankle	None	3.30	10.31	0.097	11.56	680.6	16.49
Ankle	Acc AP	**2.61**	**8.73**	**0.083**	**10.54**	614.0	15.64
Ankle	Acc ML	3.32	10.25	0.098	11.79	765.0	17.24
Ankle	Acc VT	3.69	10.67	0.105	12.20	740.1	18.51
Ankle	Ang Vel AP	2.73	9.13	0.093	11.32	**593.0**	**15.55**
Ankle	Ang Vel ML	3.68	10.37	0.102	11.67	774.9	17.81
Ankle	Ang Vel VT	3.41	10.37	0.123	12.97	677.8	16.94
Knee	None	1.96	9.77	0.046	9.98	316.6	19.00
Knee	Acc AP	1.77	9.13	**0.045**	9.74	313.3	19.54
Knee	Acc ML	**1.71**	**8.73**	**0.045**	**9.72**	296.8	18.48
Knee	Acc VT	2.14	10.21	0.053	10.86	**291.3**	**17.67**
Knee	Ang Vel AP	1.87	9.44	0.046	10.19	310.6	19.18
Knee	Ang Vel ML	1.89	9.69	0.050	10.40	273.5	17.92
Knee	Ang Vel VT	2.08	10.29	0.052	11.06	309.5	19.34

Table S6. The standard deviation across 100 Monte Carlo dropout estimations for SSL for four tissues for three characteristics.

**Structure**	**Peak (BW)**	**Impulse (BW·s)**	**Loading rate (BW/s)**
Achilles	0.579	0.0212	16.76
Patellar	0.299	0.0099	11.01
Ankle	0.778	0.0305	25.92
Knee	0.547	0.0283	21.31
